# The Washington Metropolitan Pediatric Vision Screening Quality Control Assessment

**DOI:** 10.5402/2011/801957

**Published:** 2011-12-15

**Authors:** Natario L. Couser, Janine Smith-Marshall

**Affiliations:** ^1^Virginia Pediatric Ophthalmology Specialists, Stony Point Surgery Center, 8700 Stony Point Parkway, Suite 210, Richmond, VA 23235, USA; ^2^Department of Ophthalmology, Howard University Hospital, Towers Building, Suite 2100, 2041 Georgia Avenue NW, Washington, DC 20060, USA

## Abstract

*Objective.* To ascertain if parents are familiar with current recommendations on pediatric vision screening and to assess their knowledge of the roles that pediatricians, ophthalmologists and optometrists have in this screening process. *Methods.* A survey was targeted at parents to determine what the general public understands regarding vision screening. *Results.* The survey was conducted from January–May 2010. One hundred fifty six persons responded. Over one-third did not know the difference between eye care specialists. Many believed opticians and optometrists receive medical school training. Over forty percent incorrectly identified the recommended visual acuity testing age. A large discrepancy existed regarding who should perform pediatric eye exams. Most agreed a failed screening warranted follow-up, but there was not a uniform opinion as to when to seek care. The majority of respondents understood amblyopia should be treated at least before age ten; although nine percent believed amblyopia could be treated at any age. *Discussion.* There is a significant lack of understanding of the current screening recommendations, difference between eye care professionals, and the importance of early treatment of amblyopia. *Conclusions.* Many parents do not understand the potential detrimental consequences of delayed care in the event their child fails a vision screening.

## 1. Introduction

Routine vision screening evaluations, typically performed at well child visits by the pediatrician or at school by a licensed heath care personnel, are a beneficial and cost-effective means to identify children that require care from an eye specialist [1]. Young children may be unaware and/or unable to identify problems with their vision that may be visually threatening if not diagnosed and treated [1]. Early detection and prompt treatment of ocular disorders in children are important to avoid lifelong visual impairment [2]. The American Academy of Pediatrics (AAP), the American Association for Pediatric Ophthalmology and Strabismus (AAPOS), and the American Academy of Ophthalmology (AAO) have all endorsed that vision assessments should begin at birth and that all children who are found to have an ocular abnormality or who fail vision assessments should be referred to a pediatric ophthalmologist or an eye-care specialist appropriately trained to treat pediatric patients [2]. Therefore, it is critically important that parents and care takers understand the importance of vision screening and are able to recognize the different roles of pediatricians, ophthalmologists, and optometrists. Vision loss from amblyopia is a major public health interest. Amblyopia has prevalence of 2–4% and is one of the most common causes of unilateral vision loss that if left untreated may ultimately lead to significant detrimental consequences in areas of educational achievement, sports participation, psychosocial well-being, and occupational selection [3]. It is imperative that a child with amblyopia be identified as soon as possible, as the earlier the treatment intervention can be initiated, generally the more favorable the outcome. Over 75% of amblyopic children of less than seven years of age can be successfully treated [3].

Dyslexia is a reading learning disorder. This learning disorder usually refers to a series of difficulties with reading, although individuals have normal intelligence, available educational opportunities, and social cultural chances [4]. Although its mechanisms have not been fully identified, it is thought to be affected by genetic and environmental factors, and a familial aggregation has been noticed [4]. Vision therapy has not been proven effective in treating reading problems [5]. There is a position paper endorsed by the AAP, AAPOS, and AAO which warns that vision therapy is not effective in the treatment of learning disorders. One purpose of this study is to ascertain if parents and care takers are familiar with this statement.

## 2. Patients and Methods

A 15-question survey was designed to help determine what the general public understands in regards to vision screening in children and understanding the roles of different eye care providers. Established discussion forums on high volume websites aimed at assisting, educating, and supporting parents and caretakers by facilitating open discussion were chosen to display the questionnaire to the public. The survey was anonymous but targeted at parents of the Washington Metropolitan Area. This area incorporates the District of Columbia, Northern Virginia, and parts of Maryland and West Virginia. As of the 2008 Census Bureau estimate, the population of this region was estimated to be 5,358,130, making it the ninth largest metropolitan area in the United States [6]. Reports have listed this area as one of the most educated and affluent metropolitan areas in the country [7]. Data was collected using Survey Monkey, which is a private company in the United States that allows users to create, disseminate, and analyze web-based surveys. The survey was conducted over a five-month period from January 2010–May 2010. One hundred fifty-six persons responded to the survey. Questions were formatted in a combination of nine multiple choice, four true or false, one combination-free type-in & multiple choice, and one Likert-scaling method question.

## 3. Results

### 3.1. Demographics

Demographic information collected included the age distribution of respondents, the number of children, highest level of education completed, and race. See Table 1 for demographic statistics of respondents. All respondents were 18 years or older. Only 2% were from age 18–22 years old. The majority (70%) was between ages 23 to 39 years old; 40% from age 23–29, and 30% from age 30–39. Twenty percent of respondents were from 40–49 years old. Only 9% persons were greater than 49 years old. The majority (86%) of respondents had at least one child. The largest group (60%) had one child. Twenty-one percent had two children. Those who had three children and more than three children were 4% and 1%, respectively. The respondents who completed the survey were a relatively highly educated group overall. Only 5% listed that high school was the highest level of education completed. Thirty-seven percent listed that college was the highest level of education completed. Over fifty percent listed that graduate school was the highest level of education completed. Seven people listed other; these responses were typed in as some college, medical school, vocational, still in college, doctor of medicine, massage therapy & holistic health, and additional training and certifications. Respondents represented a variety of racial backgrounds, the majority being Black or African American (50%) and Non-Hispanic White (39%). Five percent were Asian, 2% were Hispanic/Latino, and 4% listed other/multirace. 

If respondents knew the difference between an ophthalmologist, an optician, and an optometrist, and if they could identify which of the following is a physician (had completed medical school) between an ophthalmologist, optician, optometrist, and a pediatrician. A large number of respondents (35%) reported that they did not know the difference between an ophthalmologist, an optician, and an optometrist; see Table 2. They were then asked to mark those that had completed medical school among the following (multiple answers could be selected): an ophthalmologist, optician, optometrist, and a pediatrician. Over ninety percent and close to 90% checked that an ophthalmologist and pediatrician had completed medical school, respectively. However, 7% checked that an optician had completed medical school, and one-fourth of respondents checked that an optometrist had completed medical school; see Table 2.

Inquired if (1) respondents felt all children should undergo an evaluation to detect eye and vision abnormalities during the first few months of life and (2) asked about the recommended initial visual acuity testing age. Eighty-four respondents reported that all children should undergo an evaluation to detect eye and vision abnormalities during the first few months of life; see Table 3. Respondents were then asked what the recommended initial visual acuity testing age is; choices were between three years of age, five years of age, seven years of age, or only if the child complains or is observed having difficulties with vision. In response to this, the majority (59%) listed 3 years of age. Twenty-seven percent listed 5 years of age and 6% listed 7 years of age. Eight percent of respondents listed that visual acuity testing should be performed only if the child complains or is observed having difficulties with vision; see Table 3.

Who should do routine pediatric screening eye exams and visual acuity testing? The majority of respondents (over 50%) reported that pediatricians should perform routine screening eye exams and visual acuity testing. Sixteen percent reported an ophthalmologist should and 33% reported an optometrist. No respondents reported that an optician should perform the screenings. See Table 4 for the percent of overall responses regarding who respondents reported should perform pediatric screenings.

Inquired as to where respondents felt all children who are found to have an ocular abnormality or who fail vision screening should be referred to a pediatric ophthalmologist or an eye care specialist appropriately trained to treat pediatric patients; if so, what time frame should the child be seen.

overwhelmingly, (95%) respondents believed that all children should be referred to a pediatric ophthalmologist or an eye care specialist appropriately trained to treat pediatric patients if they fail a vision screening; see Table 5. There was a difference in regards to what should happen next after a vision screening has failed. The majority (76%) reported that the child should undergo a mandatory comprehensive eye exam by a licensed health care provider as soon as possible. However 5% reported the child should wait six months then have another mandatory vision screening and close to 20% thought it should be up to the parent with no mandatory regulations. See Table 5 for the percent of overall responses as to what respondents believed should happen next after vision screenings have failed.

When asked was the following response true or false: Overzealous prescribing of spectacles to infants and small children can be potentially harmful. The majority (57%) reported that overzealous prescribing of spectacles can be harmful. However, 11% reported this statement was false, and over 30% admitted that they were not sure; see Table 6.

Treatment success in amblyopia and mandatory vision screenings before kindergarten. The majority (90%) reported that the best chance for treatment success in amblyopia is before age 10. However, 1% reported before age 16, and close to 10% believed that amblyopia could be treated at any age; see Table 7. Respondents were asked how they felt about the following statement: upon entry into kindergarten, the parents or guardians of a young child must present proof of the child having passed a vision screening within the previous twelve months; there were varied responses. Forty percent strongly agreed with this statement, 42% somewhat agreed, 15% somewhat disagreed and 3% strongly disagreed; see Table 7.

Asked if learning disabilities (such as dyslexia) can be effectively treated with vision therapy. Responses were divided with slightly more persons reporting that this statement was false; see Table 8.

## 4. Discussion

Respondents in our study were a relatively well-educated group with over ninety percent completing a college degree. Eighty-six percent of the respondents were parents. The survey was posted on established discussion forums on high volume websites aimed at assisting, educating, and supporting parents and caretakers by facilitating open discussion. Therefore, in order to discover the survey link, one had to be an active participant in the discussion group. Surprisingly, over one-third of respondents admittedly did not know the difference between an ophthalmologist, optician, and an optometrist; furthermore, a significant percentage of respondents thought that an optician and optometrist receive medical school training. Sixteen percent of persons did not believe that vision assessments should begin at the first months of life which is in contradiction to current recommendations. About 40% incorrectly identified the correct Preferred Practice Pattern of recommended visual acuity testing age. Eight percent of respondents listed that visual acuity testing should be performed only if the child complains or is observed having difficulties with vision. There was a large discrepancy in who should perform routine pediatric eye exams, although the majority (over 50%) reported that pediatricians should perform routine screening eye exams and visual acuity testing. Most respondents reported that if a child fails a vision screening, they should see an eye care specialist, but there was not a uniform opinion as to when they should see the eye care specialist. The majority (76%) reported that the child should undergo a mandatory comprehensive eye exam by a licensed health care provider as soon as possible if they failed a vision screening; however, 5% reported the child should wait six months then have another mandatory vision, screening, and close to 20% thought it should be up to the parent with no mandatory regulations. Over forty percent either did not believe or were unsure that overzealous prescribing of glasses to children can be harmful. Almost 50% incorrectly thought that dyslexia could be treated with vision therapy. The majority (90%) understood that amblyopia should be treated at least before age 10; although 9% thought amblyopia could be treated at any age.

## 5. Conclusion

Currently, there is not a uniform vision screening law in the United States; screening regulations differ from state to state across the country. However, there are proposed screening guidelines and recommendations that are endorsed by the AAP, AAPOS, and AAO. There is a significant lack of understanding of the current vision screening recommendations, difference between eye care professionals, and the importance of early treatment of amblyopia. Many parents do not understand the potential detrimental consequences of delayed care in the event their child fails a vision screening. Steps should be taken to further educate the general public of the difference between eye care professionals and to promote a better understanding of the current vision screening recommendations and the importance of early treatment of amblyopia with a goal that parents will understand the importance of vision screening and the need for prompt followup if the vision screen is abnormal.

##  What Is Known, What Is New? 

(i) What is known on this subject? Routine vision screening evaluations is a cost-effective means to identify problems. Children may be unable to express problems with vision that may be visually threatening. Early detection and prompt treatment of ocular disorders are important to avoid lifelong visual impairment.

(ii) What this study adds? This study identifies the lack of understanding among parents regarding vision screening recommendations, difference between eye professionals, and the importance of early treatment of amblyopia. Steps should be taken to promote education on the current vision screening recommendations.

## Figures and Tables

**Table 1:** Demographics of respondents: age, number of children, highest level of education completed, and race.

Age	Percent of overall responses
<18 years old	0
18–22 years old	2%
23–29 years old	40%
30–39 years old	30%
40–49 years old	20%
>49 years old	9%

Total no. of responses	156

Number of children	Percent of overall responses
No children	14%
One child	60%
Two children	21%
Three children	4%
More than three children	1%

Total no. of responses	140

Highest level of education completed	Percent of overall responses
Have not completed high school	0
Completed high school	5%
Completed college	37%
Completed graduate school	54%
Other	4%

Total no. of responses	156

Race	Percent of overall responses
Asian	5%
Black or African American	50%
Hispanic/Latino	2%
Non-Hispanic White	39%
Other/multirace	4%

Total no. of responses	153

**Table 2:** Reporting knowing the difference between an ophthalmologist, an optician, and an optometrist and if they could identify which of the following is a physician (had completed medical school) between an ophthalmologist, optician, optometrist, and a pediatrician.

Reported being able to know the difference	Percent of overall responses
Yes, knew the difference	65%
No, did not know the difference	35%

Total no. of responses	153

which has had medical school training	# marked/Total Percent of those who participated in question that marked response
Ophthalmologist	143/93%
Optician	10/7%
Optometrist	38/25%
Pediatrician	134/87%

Total no. of marked/Total no. of responses	325/156

**Table 3:** Initial eye evaluation and initial formal visual acuity testing age.

Evaluation during first few months of life	Percent of overall responses
True	84%
False	16%

Total no. of responses	153

Visual acuity testing age	Percent of overall responses
3 years of age	59%
5 years of age	27%
7 years of age	6%
Only if the child has problems	8%

Total no. of responses	154

**Table 4:** Who should perform the routine screening eye exams and visual acuity testing.

Who should do routine testing	Percent of overall responses
Ophthalmologist	16%
Optician	0
Optometrist	33%
Pediatrician	51%

Total no. of responses	153

**Table 5:** Is referral necessary if an ocular abnormality is found or a vision screening failed; if a screening failed, what should happen next.

Failed screening or abnormality; should be referred?	Percent of overall responses
True	95%
False	5%

Total no. of responses	153

After failed screening, what should happen next	Percent of overall responses
Mandatory comprehensive eye exam by a licensed health care provider as soon as possible	76%
Wait six months then have another mandatory vision screening	5%
Wait one year then have another mandatory vision screening done	0
Should be up to the parent with no mandatory regulations	19%

Total no. of responses	153

**Table 6:** Overzealous prescribing of spectacles.

Overzealous prescribing of spectacles harmful?	Percent of overall responses
True	57%
False	11%
Not sure	32%

Total no. of responses	152

**Table 7:** Treatment success in amblyopia and mandatory vision screenings before kindergarten.

Best chance for treatment	Percent of overall responses
Before age 10	90%
Before age 16	1%
Amblyopia can be treated at any age	9%

Total no. of responses	152

Mandatory vision screening before kindergarten	Percent of overall responses
Strongly agree	40%
Somewhat agree	42%
Somewhat disagree	15%
Strongly disagree	3%

Total no. of responses	153

**Table 8:** If learning disabilities can be effectively treated with vision therapy.

Learning disabilities can be effectively treated with vision therapy	Percent of overall responses
True	46%
False	54%

Total no. of responses	152

## Abbreviations

AAP: American Academy of Pediatrics
AAPOS: American Association for Pediatric Ophthalmology and Strabismus
AAO: American Academy of Ophthalmology.
